# Family and community guidance in adolescence: assessment in the family health strategy^*^

**DOI:** 10.1590/1518-8345.4599.3457

**Published:** 2021-09-03

**Authors:** Rosimara Oliveira Queiroz, Márcia Moroskoski, Bianca Machado Cruz Shibukawa, Roberta Tognollo Borotta Uema, Rosana Rosseto de Oliveira, Ieda Harumi Higarashi

**Affiliations:** 1 Universidade Estadual de Maringá, Maringá, PR, Brazil.; 2 Scholarship holder at the Coordenação de Aperfeiçoamento de Pessoal de Nível Superior (CAPES), Brazil.

**Keywords:** Primary Health Care, Adolescent Health, Health Evaluation, Health Centers, Community Health Nursing, Comprehensive Health Care, Health Education, Atenção Primária à Saúde, Saúde do Adolescente, Avaliação em Saúde, Centros de Saúde, Enfermagem em Saúde Comunitária, Assistência Integral à Saúde, Educação em Saúde, Atención Primaria de Salud, Salud del Adolescente, Evaluación de Salud, Centros de Salud, Enfermería en Salud Comunitaria, Atención Integral de Salud, Educación en Salud

## Abstract

**Objective:**

to evaluate family and community guidance in adolescence, within the scope of Primary Health Care.

**Method:**

an evaluative and descriptive study with a quantitative approach, developed through the application of the Primary Care Assessment Instrument (PCATool), with 70 professionals from the Family Health Strategy and 140 adolescents from the widerange areas. Data collection took place in Basic Health Units and in the adolescents’ homes in a municipality of southern Brazil, from May to September 2019. The data were analyzed using descriptive and inferential statistics using ANOVA and Tukey’s test, performed using the R Studio software.

**Results:**

there was divergence in the assessment of the attributes of family and community guidance between users and responsible professionals, showing weaknesses in the communication process and in the formation of the care bond in this reality, with impacts on quality of care.

**Conclusion:**

there was a need for continued assessment of the care practice in primary health care, as well as for permanent education with a focus on increasing qualification of care for adolescents.

## Abstract (pt)

**Objetivo:**

avaliar a orientação familiar e comunitária na adolescência, no âmbito da Atenção Primária à Saúde.

**Método:**

estudo avaliativo, descritivo e de abordagem quantitativa, desenvolvido por meio da aplicação do Instrumento de Avaliação da Atenção Primária (*PCATool – Primary Care Assessment Tool*), junto a 70 profissionais da Estratégia Saúde da Família e 140 adolescentes das áreas abrangentes. A coleta de dados ocorreu nas Unidades Básicas de Saúde e domicílios dos adolescentes de um município do Sul do Brasil, no período de maio a setembro de 2019. Analisaram-se os dados mediante estatística descritiva e inferencial por meio da ANOVA e teste de Tukey, executados no *software* R Studio.

**Resultados:**

revelou-se divergência na avaliação dos atributos de orientação familiar e comunitária entre os usuários e os profissionais responsáveis, denotando fragilidades no processo de comunicação e na formação do vínculo assistencial nesta realidade, com impactos na qualidade da atenção.

**Conclusão:**

evidenciou-se a necessidade de avaliação continuada da prática assistencial na atenção primária em saúde, e de educação permanente com foco na qualificação crescente do cuidado ao adolescente.

## Abstract (es)

**Objetivo:**

evaluar la orientación familiar y comunitaria en la adolescencia, en el ámbito de la Atención Primaria de la Salud.

**Método:**

estudio evaluativo, descriptivo con enfoque cuantitativo, desarrollado mediante la aplicación de la Herramienta de Evaluación de la Atención Primaria (*PCATool - Primary Care Assessment Tool*), junto a 70 profesionales de la Estrategia de Salud Familiar y 140 adolescentes de las áreas incluidas. La recolección de datos se llevó a cabo en las Unidades Básicas de Salud y en el domicilio de los adolescentes de un municipio del sur de Brasil, de mayo a septiembre de 2019. Los datos se analizaron mediante estadística descriptiva e inferencial mediante el ANOVA y la prueba de Tukey, ejecutados en el software *R Studio*.

**Resultados:**

se encontraron diferencias en la evaluación de los atributos de la orientación familiar y comunitaria entre los usuarios y los profesionales responsables, demostrando la existencia de debilidades en el proceso de comunicación y en la formación del vínculo asistencial, que impactan en la calidad de la atención.

**Conclusión:**

existe la necesidad de evaluar continuamente la práctica asistencial en la atención primaria de salud y de educar permanentemente a los profesionales para mejorar su cualificación para atender a los adolescentes.

## Introduction

The adolescence phase is associated with several milestones and peculiarities, being defined as the period of life that individuals enter when they turns 12, corresponding to a stage of transition from childhood to adulthood. In this process, adolescents face a series of changes in physical, cognitive, psychosocial, sexual and identity crises. The need for autonomy and the discovery of identity with family and society are also common^(1-2)^.

Within the scope of Primary Health Care (PHC), several gaps or difficulties still permeate actions aimed at the adolescents, which include from lack of training of the professionals, including structural deficiencies in health units for the reception of the adolescents to lack of involvement of this clientele in the action planning process, thus composing a scenario of weaknesses that compromises comprehensive quality care^(3-5)^.

Therefore, the challenges of the Family Health Strategy (FHS) demand greater political and institutional effort, through financing, personnel management and education and the guarantee of comprehensive care, with a view to achieving the appropriate balance between the individual approach in a timely manner and the community approach to address the social determinants, challenges that hinder the organization of PHC as the main axis of the health system^(4,6-7)^.

Therefore, adolescents’ adherence to primary care actions, especially in the FHS, is stimulated by structuring the service, preparing the professional and the team, and by the adolescent understanding the health-disease processes^(4,6)^. In this sense, it is worth mentioning that the work process of the FHS, largely aimed at specific audiences and programs, can be the main responsible for the reductionist knowledge of their actions, by the adolescent and by other users of the service^(8)^.

Family and community guidelines stand out as tools that promote greater interaction between the FHS and the family and community^(9)^. The importance of the affective bond and the trust established by the user and the health professional are emphasized as factors that enable comprehensive health care for the population^(10)^.

It is evidenced that the actions developed by nurses for adolescents are based on guidelines, often in partnership with the school, for better effectiveness in the connection with this population, as the professionals face the absence of adolescents in the health service^(4-8)^.

Generally, the demand for health services by adolescents is imposed by parents or guardians, and not motivated by an intimate decision; nor are adolescents allowed a more active participation in care, restricting the communication process between the adolescent and the health professional, since feelings of shyness or embarrassment are common^(11)^.

Another strategy used by FHS teams to reach adolescents is home visits, promoting health and care practices permeated by trust and bond between professionals and adolescents, executing family and community guidelines^(12)^.

However, through a general survey of PHC services worldwide, assessed with the PCATool instrument, it was found that the family, community and other attributes present weaknesses. Most of the studies surveyed in this review were carried out in Brazil, which highlights the need to strengthen the different components that make up the PCATool, as a way to improve the performance of PHC in our country^(13)^.

Therefore, carrying out the assessment of family and community guidelines allows for the creation of subsidies capable of contributing to the qualification of assistance, emphasizing the communication process, so important in the care context of this clientele. However, the hypothesis arises that the family and community guidance provided by the FHS for adolescents present weaknesses.

Through this context, the objective was to assess family and community guidance in adolescence, within the scope of Primary Health Care.

## Method

This is an evaluative, descriptive and quantitative research study, developed through the application of the Primary Care Assessment Instrument (PCATool), child and professional versions, assessing the Family and Community Guidance attributes. To guarantee the methodological quality of this study, the recommendations of the Enhancing the Quality and Transparency of Health Research (EQUATOR) Network were used through The Strengthening the Reporting of Observational Studies in Epidemiology (STROBE) checklist^(14)^.

The study was carried out in Maringá, a municipality located in the state of Paraná, southern Brazil. Data collection took place from May to September 2019.

The population of this study was composed of adolescents aged 12 to 18 years old, as defined in the Child and Adolescent Statute^(1)^ and of the FHS nurses. The option to select these team members was due to the fact that they are leading professionals in the FHS team and have a greater bond with the population.

For sample calculation, the adolescents were randomly selected, aiming to represent all the areas covered by the FHS. The study municipality has 77 FHS teams, each one having a nurse, with seven teams not being covered by this category; which is why they were excluded from the study.

In the case of independent samples with different sizes, the combined α was used, representing the estimate of the aggregated standard deviation of the samples, *n*1 − 1 and *s1²*, corresponding to the degree of freedom and variance of the first group, *n*2 − 1 and *s2²* both from the second group. The final sample consisted of 140 adolescents and 70 FHS nurses, through prior contact and scheduling of day and time with the nurses, adolescents and their guardians. For the interview, the researcher visited the nurses‘ workplace and the adolescents’ homes, and applied the Primary Care Assessment Tool (PCATool) Brazil, child and professional versions^(15)^.

The instrument assesses the attributes of family and community guidance for professionals and adolescents. The answers of this instrument followed a Likert scale, with a score from 1 to 4 (certainly yes = 4; probably yes = 3; probably no = 2; certainly no = 1)^(15)^.

For data analysis, the scores of the attributes of family and community guidance of the adolescents and professionals were computed by averaging the values of the answers of the items that compose them, being subsequently transformed into a scale from 0 to 10 by means of the formula recommended by the research instrument: [score obtained – 1] x 10 divided by 3. Scores ≥ 6.6 were classified as high, which corresponds to a value of three or more on the Likert scale and those <6.6, as low^(15-16)^.

To determine the power of the analysis, the “*pwr*” package of the *R Studio* software was used. Fixing the sample size (70 nurses and 140 adolescents), the effect size (0.5) and the significance level (5%), a power of approximately 93% was obtained.

The Analysis of Variance test was performed followed by the use of the Tukey test to compare individual means and, in the ANOVA test, a significance level of 5% was adopted to compare the mean scores of the answers by the adolescents and health professionals.

The measures of the effect size are defined at three levels, calculated according to “Cohen’s d” in which the value is small if 0*.*20 *< d <* 0*.*50, medium if 0*.*50 *< d <* 0*.*80 and large if 0*.*80 *< d* ^(17)^. For this study, mean sizes of d = 0.5 were used since research was not feasible for smaller values. All the analyses were performed in R Studio-version: 1.2.5001-2019.

The study followed all the ethical and legal precepts contained in Resolutions 466/2012^(18)^ and 510/2016^(19)^ of the National Health Council. The research was approved by the Standing Committee on Ethics and Research with Human Beings of the State University of Maringá, under CAAE No. 10627519.1.0000.0104 and report No. 3,266,229. All the participants were informed about the study and signed the Free and Informed Consent Form and the Free and Informed Assent Form.

## Results

The analysis of the results obtained through the application of the PCATool instrument with the 140 adolescents and 70 professional nurses from the FHS who were interviewed revealed dissenting opinions in both attributes of family and community guidance. Thus, while the professionals consider adequately fulfilling the guidance assignments recommended in the instrument, the adolescents reveal that they do not perceive such actions.

With regard to the family guidance attribute, it is emphasized that the highest percentage of divergence in the answers between adolescents and professionals referred to the “I1 - Does your doctor/nurse ask you about your ideas or opinions about your treatment?” question, in which the adolescents answered that they did not receive this type of questions during their care (82.86%). For the same question, however, the professionals claimed that they indeed usually ask this type of questions during consultations (71.43%).

Also in the family guidance component, a gap is evidenced since, in the adolescents’ perception, the FHS professionals do not ask about the problems and diseases of their family members and a little more than half of the adolescents do not believe that the professionals would meet with family members to discuss some type of health problem, as shown in Table 1.

**Table 1:** Family guidance in the perception of adolescents and professionals of the Family Health Strategy in a municipality of the South of the country. Maringá, PR, Brazil, 2020

Answers	Adolescent					Professional				
	Yes		No		Total	Yes		No		Total
Questions	N	%	N	%	N	N	%	N	%	N
I1*/G1^†^	24	17.14	116	82.86	140	50	71.43	20	28.57	70
I2^‡^/G2^§^	49	35.00	91	65.00	140	69	98.57	1	1.43	70
I3^||^/G3^¶^	70	50.00	70	50.00	140	69	98.57	1	1.43	70

* I1 = Does your doctor/nurse ask you about your ideas or opinions about your treatment?;

† G1 = Do you ask the patients what their ideas and opinions are when planning the treatment and care of the patient or family member?;

‡ I2 = Has your doctor/nurse asked you about diseases or problems in your family?;

§ G2 = Do you ask about diseases and health problems that may occur in the adolescents' families?;

|| I3 = Your doctor/nurse would meet with other members of your family if you felt it necessary;

¶ G3 = Are you willing and able to assist family members of adolescents to discuss a health or family problem?

Similarly, the same type of disagreement between the answers was found in community guidance, with the “J4 - Some health professional invites family members to participate in the local health council (Management council/User council)” question standing out, in that the adolescents denied receiving the invitation (79.29%), in contrast to the professionals, who reported carrying out the invitation (71.43%).

**Table 2:** Community guidance in the perception of adolescents and professionals of the Family Health Strategy in a municipality of the South of the country. Maringá, PR, Brazil, 2020

Answers	Adolescent					Professional				
	Yes		No		Total	Yes		No		Total
Questions	N	%	N	%	N	N	%	N	%	N
J1*/H1^†^	73	52.14	67	47.86	140	68	97.14	2	2.86	70
J2^‡^/H2^§^	69	49.29	71	50.71	140	61	87.14	9	12.86	70
J3^||^/H5^^¶^^	30	21.43	110	78.57	140	34	48.57	36	51.43	70
J4**/H6^††^	29	20.71	111	79.29	140	50	71.43	20	28.57	70
H3^‡‡^	-	-	-	-	-	63	90.00	7	10.00	70
H4^§§^	-	-	-	-	-	39	55.71	31	44.29	70

* J1 = Does someone from the health service/or doctor/nurse make home visits?;

† H1 = Do you or someone in your health service make home visits?;

‡ J2 = Do health professionals know the important health problems in your neighborhood?;

§ H2 = Do you believe that your health service has adequate knowledge of the health problems of the community it serves?;

|| J3 = Does any health professional does research to identify health problems that they should know about?;

¶ H5 = Does he do research in the community to identify health problems that he should know about?;

** J4 = Some health professional invites family members to participate in the Local Health Council (Management Council/User Council);

†† H6 = Presence of users on the Local Health Council (Management Council/Users Council)

‡‡ H3 = Does your health service hear community opinions and ideas on how to improve the health services?;

§§ H4 = Do you do patient research to see if the services are satisfying (meeting) people's needs?

With regard to community guidance, the role of home visits stands out, with performance reported by 52.14% of the participating adolescents and 97.14% of the health professionals. Nevertheless, they pointed to deficiencies in the implementation of community guidance, explained by lack of investigation of health problems, mentioned in 78.57% by adolescents and in 51.43% by professionals (Table 2).

Given these answers, when calculating the attribute scores according to the instructions of the PCATool^(12)^ instrument, it was verified that the professionals obtained higher scores in both types of guidance, family (7.89) and community (6.99), thus punctuating the effective accomplishment of the guidelines. However, the scores of the adolescents’ answers were low for family (3.28) and community (3.64) guidance, characterizing the guidelines as ineffective, according to the perception of these users; communication process between professional/service and user.

Given the results of the assessment of family and community guidance found in the present study, with significantly low scores in the adolescents in contrast to the scores of the professionals, which were significantly high, it is possible to infer an important deficit in the communication process between these two populations, as shown in Table 3.

**Table 3:** Scores of the family and community guidance attributes according to the answers of the adolescents and the professionals from Family Health Strategy in a municipality of the South of the country. Maringá, PR, Brazil, 2020

	Adolescent		Professional	
	Family member	Community	Family member	Community
Mean	3.28	3.64	7.89	6.99
Standard Deviation	2.55	2.57	1.37	1.72
Median	3.3	3.3	7.78	7.22
Maximum	10	10	10	10
Minimum	0	0	4.44	1.67

**Table 4:** Comparison analysis of the mean scores of the attributes of community and family guidance according to the analysis of variance (ANOVA) test. Maringá, PR, Brazil, 2020

	Degree of freedom	Sum of squares	Mean sum of squares	F value	p-value (>F)*
Guidance (Fam^†^/Com^‡^)	1	0.41	0.41	0.080	0.7775
Group (Prof^§^/Ado^||^)	1	1,480.83	1,480.83	2,861,904	<2.2e- 16
Guidance: Group	1	36.65	36.65	70,832	<0.0081
Residuals	416	2,152.50	5.17	-	-

* p-value(>F) = Significance level;

† Fam = Family members ;

‡ Com = Community;

§ Prof = Professionals ;

|| Ado = Adolescent

It is worth highlighting the comparison of the mean scores of the groups with the analysis of variance (ANOVA), which showed significant differences between the mean scores for professional/guidelines and adolescents/ guidelines, evidencing the disagreement between them about family and community guidelines, obtaining a p-value <2.2 e-16 and <0.0081 respectively, considering a significance level of 5% (Table 4).

For a detailed comparison of these differences obtained in the Tukey test, eight combinations were made: family guidance/community guidance; professional/ adolescent; family guidance (adolescents)/community guidance (adolescents); family guidance (professionals)/ community guidance(adolescents); community guidance (professionals)/community guidance (adolescents); family guidance (professionals)/family guidance (adolescents); community guidance (professionals)/family guidance (adolescents); family guidance (professionals)/ community guidance (professionals).

**Table 5:** Multiple comparisons between the mean scores of the professional and adolescent groups. Maringá, PR, Brazil, 2020

Variables	^‡^	CI^§^	p-value^||^
F.G.*/C.G.^†^	0.0628	0.37-0.50	0.7775
Professional/Adolescent	3.9832	3.52-4.45	<0.001
F.G.* Adolescent/C.G.^†^ Adolescent	0.3550	1.06-0.35	0.5598
F.G.* Professional/C.G.^†^ Adolescent	4.2549	3.40-5.11	<0.001
C.G. Professional/C.G.^†^ Adolescent	3.3566	2.50-4.22	<0.001
F.G.* Professional/F.G.* Adolescent	4.6099	3.75-5.47	<0.001
C.G. Professional/F.G.* Adolescent	3.7116	2.85-4.58	<0.001
F.G.* Professional/C.G. Professional	0.8983	0.09-1.89	0.0916

* F.G. = Family guidance;

† C.G. = Community Guidance;

‡ Difference of means

§ IC = Confidence Interval;

|| p-value = Significance level

The combinations show the lack of agreement between adolescents and professionals in both community and family guidelines, with agreement only within the same group, as shown in Table 5. Thus, through the results presented, the hypothesis of the presence of fragility in the family and community guidance performed for adolescents by the FHS is confirmed.

## Discussion

The results of this study show weaknesses in the communication and bonding process between FHS professionals and adolescents in the context of PHC, in view of the discordant results in the assessment of the attributes of community and family guidance between these two groups.

The attributes of family and community guidelines are recognized as those that portray the greatest interaction of the FHS with the family and the community^(9)^. Thus, the importance of the affective bond and the trust established between professionals and adolescents is emphasized, factors that facilitate comprehensive health care for the population^(10,20)^.

The lack of protagonism of the adolescents in their own consultations, in which care is taken on by the responsible companions, constitutes a challenge in the process of building adolescents’ health autonomy^(3)^.

Such obstacles are often represented by an inflexible attitude of parents or guardians, when they do not consider the opinion of the adolescents, in various aspects of life and, mainly, in matters related to their health^(7,21)^. In this way, it is possible to explain the nurses’ statements in the family and community guidelines, since they did not perceive the adolescent as the protagonist of the guidelines, but their guardians.

The commitment to the adolescents is essential for effective health care, as well as the formation of appropriate care and decision-making behaviors^(22)^. Therefore, health professionals and services need to adopt a welcoming attitude towards this clientele and actions that encourage adolescents’ autonomy in their transition to adult life, thus strengthening the attributes of family and community guidance in the context of PHC^(4,22)^. It is also reinforced that the adherence of the adolescents to the health services is linked to the method used for their recruitment, the interest of the multidisciplinary team and in the dissemination of actions^(23)^.

In this scenario, the adolescents’ greater interest in topics related to health and quality of life is evidenced. Thus, in addition to not having contact with the topic at home, and needing to seek knowledge in other ways, the adolescents refer to ignoring the existence of programs aimed at adolescent health in the place where they live, which shows the lack of initiatives of this nature in the list of services offered to these individuals^(24)^.

The adolescents in this study denied the presence of a link with PHC, which invariably affects the adolescent’s weakened relationship with their health. This situation contributes to the formation of uninformed adults and little committed to care and health services, which, ultimately, is reflected in the increase in queues at hospitals, specialties, surgeries and even in premature death from preventable causes^(6-7)^.

Understanding adolescents as autonomous subjects does not imply leaving them out of protective measures or political protection actions, but rather seeking to guarantee their inclusion in the planning process of their health promotion actions, understanding the specificity of the phase experienced and the peculiarities of each individual^(25-28)^.

In addition to the challenge of understanding and establishing bonds with the adolescents, it is necessary to ensure the fundamental assumption of respect for the individual, at any age, as a citizen or future citizen, with the right to dignity. Such condition, extended to the adolescent population, and provided for in the public policies, should seek to guarantee, among other aspects, autonomy with respect to their own health condition^(21)^.

Another aspect of an ethical nature that stands out in the assessment of the adolescents is in relation to confidentiality during health consultations. In this sense, it is recommended to provide assistance in two stages or moments, one only with the adolescent and the other, in the presence of their guardian, when necessary^(22)^. Breach of confidentiality, in turn, must represent a case of exception, and be restricted only to the cases provided for in the professional code of ethics^(22-26)^.

Health care must include all biopsychosocial aspects of the adolescent’s life; therefore, family guidance must always be encouraged, seeking to also consider the family context. A study points out that, when nurses attach importance to the inclusion of families in Nursing care after an educational intervention, there are positive impacts on the care practices^(10,27)^.

The home visit was reported as present in this study, but without in-depth investigation when performed. The home visit of the FHS must aim to monitor at-risk and vulnerable groups, with the perspective of promoting health and preventing diseases, so that, for these groups, visits are made with a mean monthly periodicity^(27)^.

Home care provides professionals and the service an adequate diagnosis of the problems and needs of the communities served, in addition to providing an approximation to the aspects related to the family structure and the home infrastructure, expanding the nurses’ understanding of this clientele, making it possible to avoid unnecessary hospitalizations and quick action in the face of the health demands^(28)^.

It is worth mentioning that, within the FHS, community health agents are fundamental in the organization of the care model, and should therefore be included in care planning, from population diagnosis activities, to the needs that emerge in the community, and implementations of health actions, assisting in social participation^(29)^.

Community knowledge is essential for the construction of the situational diagnosis, which enables the development of actions and planning according to the needs of the community, in addition to active listening dialogic actions involving professionals and users for care planning^(30)^.

The lack of effective communication between services (represented by the FHS professionals) and users (represented by the adolescents) found in this study is quite worrying. There is an urgent need for investments aimed at strengthening these attributes of PHC, which represent the foundation of the link between community and service, and a cornerstone for the success of the health promotion process for this clientele^(20,22)^.

The work with adolescents still represents a great challenge for FHS nurses, since it is a clientele that hardly uses the health service. In addition, the lack of development of specific actions for this population is motivated by the lack of structure and resources, in addition to the work overload in the teams^(2,22-25)^.

Another evident challenge is the obstacles present in serving this population, such as the lack and the inappropriate physical structure of the units, with negative implications for holding meetings with the adolescents, and also the high demand for teamwork, thus hindering to conduct educational practices^(8-9,11,21)^.

Group intervention for adolescents needs to be a priority in the FHS; however, the actions to promote, prevent and recover the health of this group need to be strategically planned, since the adolescent’s adherence is linked to the method used, the interest of the multidisciplinary team and in action disclosures^(13,31)^.

Worldwide, the PHC services that were assessed using the PCATool instrument demonstrated that the family and community guidance attributes, as well as others, present weaknesses^(21-36)^. In Brazil, the scenario is no different, which highlights the need to strengthen the different components that make up the PCATool, as a way to improve PHC performance in our country^(37-40)^.

The international literature also points out that both in developed and in developing countries, actions aimed at adolescent health are fragmented, poorly coordinated and of irregular quality, in addition to the fact that the professionals are unprepared to work according to the adolescents’ behavior and language. Such factors impair the insertion of adolescents in primary health care, preventing literacy in adequate health, which can generate invaluable losses for this group that is already vulnerable^(41-42)^.

Despite the recognition of the importance of PHC in organizing care for being the gateway to health care, there is still a need for investment to improve attributes, so that PHC fulfills its assistance as recommended^(39-40)^, assisting adolescents in health literacy, regarding disease prevention and treatment; as well as health promotion measures, attitudes that promote economic and social benefits, in addition to meeting the requirements of the World Health Organization for global health convergence by 2030^(43)^.

In view of these circumstances, it is up to the health services to review their attitudes and concepts about the care process and the evaluation of the service provided to adolescents. In this sense, the evaluation must be supported by the understanding that any negative results must be approached as effective contributions to the improvement of the system, allowing the professionals to reflect on their actions, promoting planning and the decision-making process, aspects that help to increasingly qualify the assistance provided^(40)^.

Thus, it is relevant for the improvement of assistance in adolescence to incorporate national guidelines for health care for adolescents and young people, in the demands for this population, which indicate adopting planning and health promotion actions that have as their articulation center the adolescents taking into account their life projects, as well as their family and economic sociocultural context^(44)^.

It becomes necessary to include the adolescents in the construction of health plans and actions so that they become more involved with their health, thus guaranteeing their autonomy and supporting the work of the FHS. In addition, sensitivity to the demands and needs of this population is recommended, emphasizing their individual, social, ethnic and territorial diversity, establishing partnerships with cultural, sports activities and schools that operate in the same territory^(11,21,23,25,44)^.

In addition, putting into practice the Health in the Schools Program (*Programa Saúde nas Escolas*, PSE), which is a program created by the Brazilian government with the objective of contributing to the integral education of students through health promotion, disease prevention and health care actions^(45)^. Some studies have pointed out the importance and the benefit of the PSE in actions in health promotion and in the construction of the bond between adolescents and health professionals. In this way, the FHS team establishes the bond of trust and respect, the essential factor for effective assistance to this population^(46-47)^.

The importance of the knowledge constructed by the study is highlighted to foster changes in the context of the assistance provided to adolescents, which must be thought for the adolescent in a participatory manner. It is necessary to develop autonomy in health, exercising the role of assistance and emphasizing the relevance of evaluating the service as an exercise of the democratic right to guarantee quality of care for adolescents.

Finally, it is worth mentioning as limitations of this study that many programs and actions are not designed to be evaluated, presenting little exact information about all the expected points, hindering its implementation as well as subsequent evaluation. Another limiting factor was the FHS teams not covered by the nurse, making it impossible to collect data in its wide-range area, in addition to the difficulty in finding other studies aimed at assessing PHC in the area of adolescent health care.

## Conclusion

The attributes of family and community guidelines were negatively evaluated by the adolescents attended by the FHS; however, from the nurses’ perspective, both attributes were considered satisfactory. This discrepancy leads to two important concerns: the first, related to the importance of stimulating the process of continuous and coherent evaluation, free from biases that prevent the proposal of changes in the practices and, therefore, improvement of the system and qualification of care; and the second, related to an evident gap and inefficiency of the communication process between community and service, between users and professionals.

Thus, and considering the importance of the attributes of family and community guidance as precursors and facilitators for the formation of the bonds of trust fundamental to the care and therapeutic process in health, the need is evidenced for all who make up the FHS team to strive so as to strengthen these PHC components in order to favor the promotion, prevention and recovery of adolescent health.
